# Metabolic Regulation of Cardiac Differentiation and Maturation in Pluripotent Stem Cells: A Lesson from Heart Development

**DOI:** 10.31662/jmaj.2020-0036

**Published:** 2020-07-13

**Authors:** Yuika Morita, Shugo Tohyama

**Affiliations:** 1 Department of Cardiology, Keio University School of Medicine, Tokyo, Japan; 2 Department of Organ Fabrication, Keio University School of Medicine, Tokyo, Japan

**Keywords:** Metabolism, Pluripotent Stem Cells, Cardiac differentiation, Heart Development, Regenerative Medicine

## Abstract

The heart, one of the more complex organs, is composed from a number of differentiated cells. In general, researchers consider that the cardiac cells are derived from the same origin as mesodermal cells, except neural crest cells. However, as the developmental stages proceed, cardiac mesodermal cells are differentiated into various types of cells via cardiac progenitors and demonstrate different programming in transcriptional network and epigenetic regulation in a spatiotemporal manner. In fact, the metabolic feature also changes dramatically during heart development and cardiac differentiation. Researchers reported that each type of cell exhibits different metabolic features that can be used to specifically identify them. Metabolism is a critical process for generating energy and biomass in all living cells and organisms and has been long regarded as a passenger, rather than an active driver, for intracellular status. However, recent studies revealed that metabolism influences self-renewal and cell fate specification via epigenetic changes directly or indirectly. Metabolism mirrors the physiological status of the cell and endogenous cellular activity; therefore, understanding the metabolic signature of each cell type serves as a guide for innovative methods of selecting and differentiating desired cell types. Stem cell biology and developmental biology hold great promise for cardiac regenerative therapy, for which, successful strategy depends on the precise translation of the philosophy of cardiac development in the early embryo to the cell production system. In this review, we focus on the metabolism during heart development and cardiac differentiation and discuss the next challenge to unlock the potential of cell biology for regenerative therapy based on metabolism.

## Introduction

**Figure 1.**
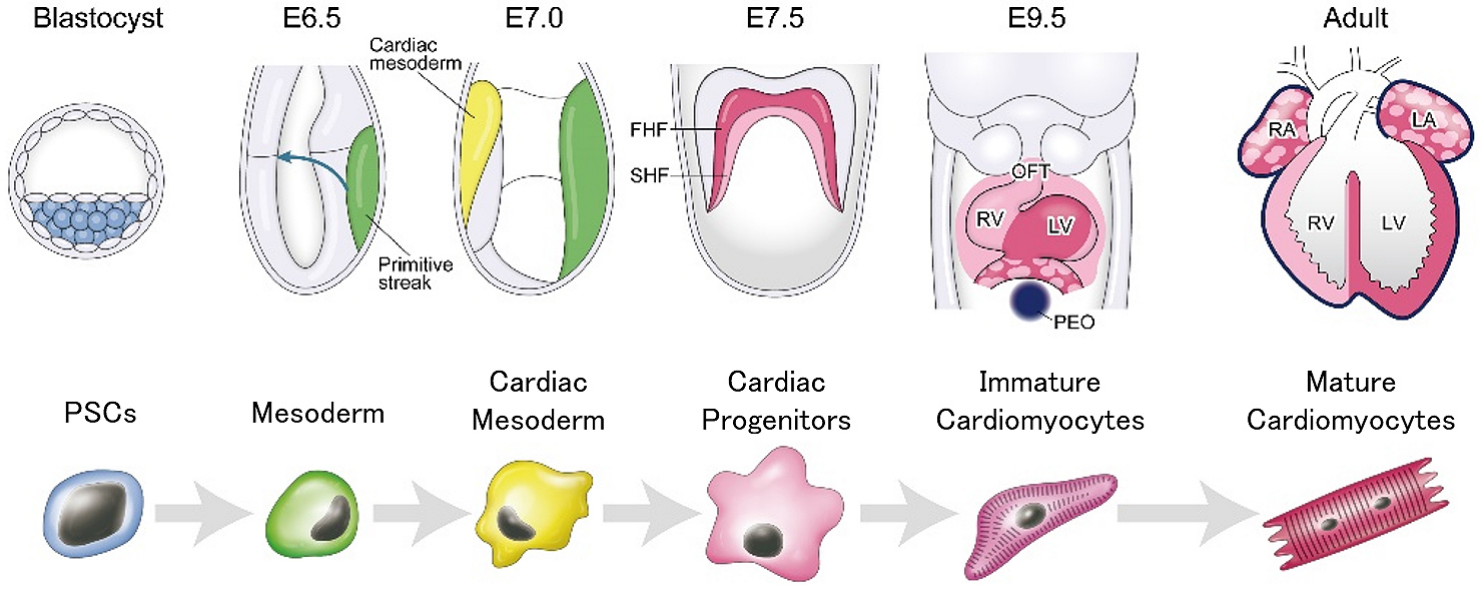
Schematic representation of heart development and cardiac differentiation. In the blastocyst stage, inner cell mass (ICM, blue) gives rise to three germ layers: ectoderm, endoderm, and mesoderm, and generates definitive structure of fetus. At embryonic day 6.5 (E6.5), endoderm and mesoderm layers emerge from primitive streak and migrate bilaterally. At E7.0, mesodermal cells migrate into the anterior region and generate cardiac crescent. At E7.5, first heart field (FHF, red) and second heart field (SHF, pink) are formed. By E9.5, looped heart tube is formed and outflow tract (OFT), right ventricle (RV) and left ventricle (LV), proepicardial organ (PEO, purple), etc. are formed. LV is derived from FHF, OFT and RV are mainly derived from SHF, and both the aria are derived from FHF and SHF. The colors are correlated with the stages in the cardiac differentiation.

**Figure 2.**
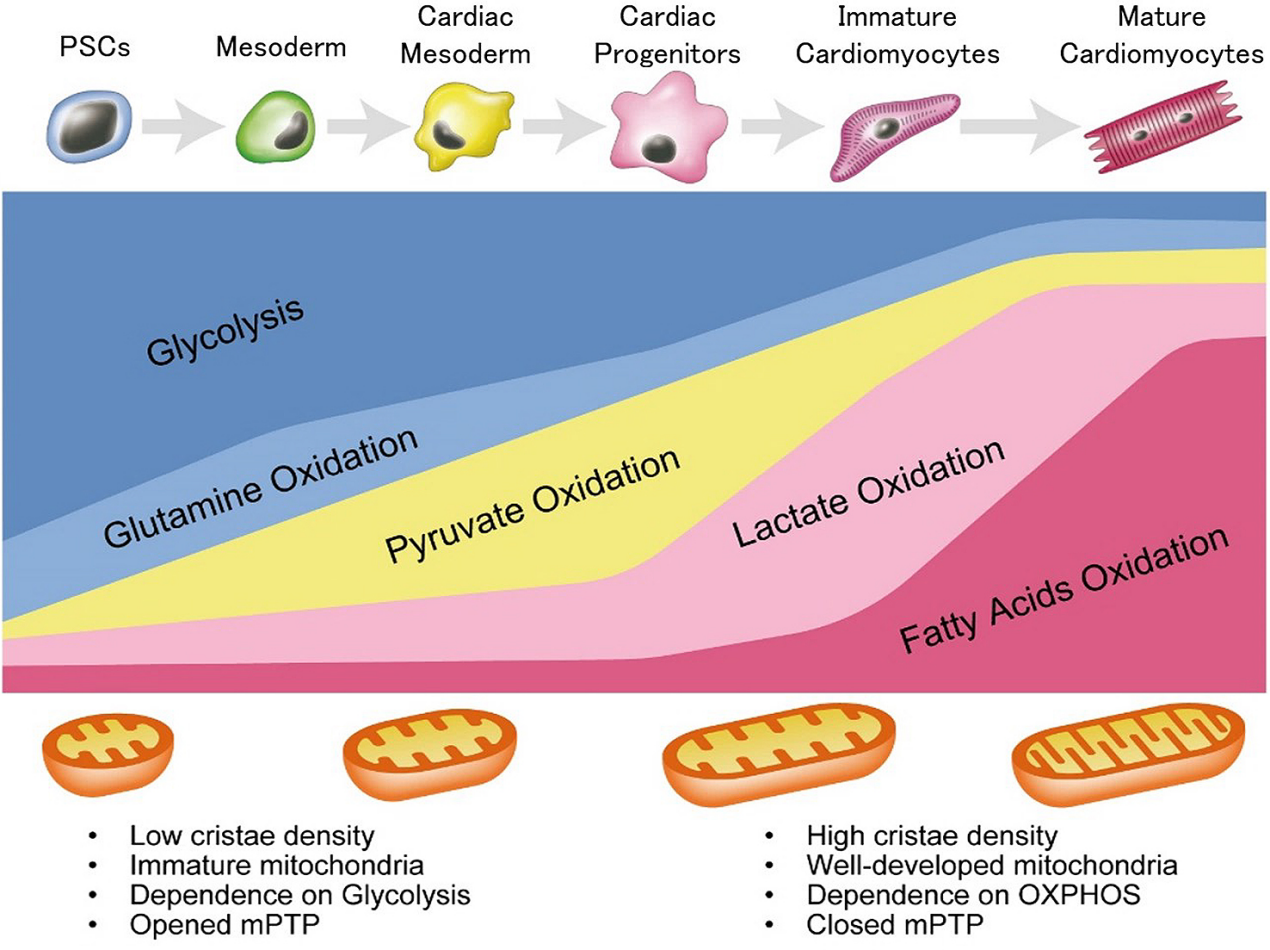
Stage-specific metabolic feature during cardiac differentiation from pluripotent stem cells. PSCs mostly depend on glycolysis and glutamine oxidation metabolism. They exhibit immature mitochondria with low cristae density. During mesoderm and cardiac mesodermal stage, these cells still mainly depend on glycolysis and glutamine oxidation; however, pyruvate oxidation metabolism is also activated as mitochondrial development proceeds. In cardiac progenitor stage, these cells depend on aerobic and anaerobic metabolism equally. After differentiation into cardiomyocytes, mitochondrial number is increased and mitochondrial maturation is proceeded. Mitochondrial permeability transition pore (mPTP) is closed, when cardiac differentiation proceeded. mPTP closure enables the mitochondrial elongation and induces higher mitochondrial membrane potential. These cardiomyocytes depend mainly on lactate oxidation metabolism and fatty acid oxidation metabolism.

Pluripotent stem cells (PSCs), including embryonic stem cells (ESCs) and induced pluripotent stem cells (iPSCs), are expected to be useful for cell-based therapy and pharmacological screening, owing to their ability for theoretically infinite production of required cell types including cardiomyocytes. For realization of cell-based therapy and pharmacological screening, pure and matured cardiomyocytes derived from human PSCs (hPSCs) are needed in large quantities. To obtain large quantities of pure and matured cardiomyocytes, development of efficient cardiac differentiation, purification, and maturation protocols is necessary. For the development of efficient cardiac differentiation protocols, complicated signaling in heart development was studied because processes of cardiac differentiation from PSCs are known to be similar to those of heart development (Figure 1) ^(1), (2), (3), (4)^. In cardiomyocyte purification, combinations of fluorescence activated cell sorting (FACS) and genetic modification or the use of antibodies for cardiomyocyte-specific surface proteins (e.g. VCAM-1, SIRPA) are major strategies ^(5), (6)^. However, due to the use of FACS, these strategies are not suitable for large-scale production of cardiomyocytes. In cardiac maturation, physical interventions, including tissue engineering and electrical stimulation, are known to be effective ^(7), (8)^. Conversely, many studies recently demonstrated that metabolic regulation also affects the survival, maintenance, and differentiation of PSCs ^(9), (10)^. Also, our group developed a large-scale metabolic selection system for hPSC-derived cardiomyocytes, based on developmental metabolic profiles ^(11), (12), (13), (14)^. These studies suggest that a better understanding of developmental metabolism demonstrates the potential to provide an alternative solution for improvement of cardiac differentiation, purification, and maturation in PSCs (Figure 2). Cellular metabolism was recognized as a passenger for PSCs and differentiated cells for proliferation, self-renewal, and differentiation. However, this concept is changing in that cell metabolism is one of the drivers for cell fate decision ^(15)^. Additionally, understanding cell metabolism may provide new insights on the phenomena of proliferation, differentiation, and maturation. Here, we review the metabolic shift as a key driver during heart development and cardiac differentiation.

## Metabolism in Blastocyst and Pluripotent Stem Cells

The blastocyst is composed of two distinct cell lineages: inner cell mass (ICM), which gives rise to definitive structures of fetus, and trophectoderm (TE), which predominantly forms extraembryonic lineages. The TE contains a greater number of mitochondria than ICM, consumes oxygen, and activates mitochondrial oxidative phosphorylation (OXPHOS) ^(16)^. Researchers considered that TE generates approximately 80% of ATP, which is mainly used for the sodium pump (Na^+^, K^+^, ATPase) located in the TE ^(16)^. In contrast, glycolysis is activated, and a lot of lactate is produced in ICM (Figure 1) ^(17), (18)^. ICM shows low mitochondrial membrane potential, indicating low levels of OXPHOS ^(19)^.

Amino acids are also known to be key molecules for proper blastocyst development ^(20)^. Interestingly, TE or ICM alone extracted from bovine blastocyst shows that amino acid turnover is dramatically increased. Furthermore, TE and ICM exhibit different amino acid profiles for consumption and production, respectively ^(21)^. In the bovine blastocyst, aspartate, glutamate, and arginine are consumed, while alanine is produced ^(21)^ Whereas, in ICM extracted from bovine blastocyst, aspartate, asparagine, glycine, threonine, arginine, tyrosine, tryptophan, leucine, and lysine are consumed, while glutamate, serine, histidine, glutamine, alanine, methionine, valine, and isoleucine are produced ^(21)^. In TE, leucine and arginine are preferably utilized in bovine, mice, and humans ^(21), (22), (23)^. Leucine and arginine alone are necessary and sufficient for mouse trophoblast outgrowth, and mTOR activation and protein translation are necessary for trophoblast outgrowth by these amino acids. This result suggests that leucine and arginine help in the attachment of trophectoderm to uterine epithelial cells to initiate the implantation ^(23)^. Besides, methionine metabolism is crucial for blastocyst development ^(24), (25)^. Methionine deprivation caused a reduced H3K4 methylation and altered gene expression profiles. Therefore, maternal methionine restriction induced embryonic lethality in mice ^(25)^.

Fatty acid metabolism also demonstrates a key role in blastocyst development. Octanoate, a medium-chain fatty acid, when incorporated into TCA cycle, produced ATP via β-oxidation process ^(26)^. Although blastocysts could not normally develop under culture conditions without fatty acids, glutamine, and pyruvate, octanoate supplementation can rescue blastocyst development, indicating that blastocysts utilize fatty acids for energy production, and octanoate can provide alternative energy source through preimplantation development ^(26)^.

As PSCs constantly self-renew and proliferate *in vitro*, protecting them from reactive oxygen species (ROS) via OXPHOS is crucial for maintaining genome stability because ROS induces nuclear and mitochondrial DNA damage, protein oxidation, and lipid oxidation. Recent studies showed that hPSCs exhibits different metabolic characters depending on pluripotency state, and these differences demonstrate an influence on distinct differentiation potential ^(27)^. Nevertheless, similar to embryo development, mouse and human PSCs mainly depend on glucose and glutamine metabolism (Figure 2) ^(12), (28)^. This preference makes sense in suppression of ROS generation for PSCs. Naïve mouse PSCs (mPSCs) utilize glucose and glutamine to maintain high α-ketoglutarate (αKG) levels because elevated αKG promotes histone/DNA demethylation and contributes to the maintenance of pluripotency ^(29)^. In addition, glucose and glutamine metabolism contributes to both ATP generation and biomass production in hPSCs ^(12), (29), (30)^. Interestingly, glycolytic genes precede pluripotent marker genes in the reprogramming process in mPSCs ^(30)^. Moreover, glucose-derived cytosolic acetyl-CoA promotes histone acetylation under pluripotent state in mouse and human PSCs ^(31)^. To keep pluripotency in mouse and human PSCs, histone acetylation also demonstrates a pivotal role in the maintenance of an open chromatin structure ^(32), (33), (34), (35)^. Furthermore, glutamine metabolism also contributes to maintain pluripotency because glutamine-derived reduced glutathione prevents degradation of OCT4. Among the other amino acids, threonine and methionine contributes to S-adenosylmethionine (SAM) production in mouse and human PSCs, respectively, leading to subsequent trimethylation of histone H3 lysine 4 (H3K4me3) for the maintenance of pluripotency ^(19), (36)^. Researchers also reported that *de novo* fatty acid synthesis regulated by lipogenic enzyme ACC1 promotes reprograming of human fibroblasts by affecting mitochondrial fission ^(37)^.

## Metabolism in Mesoderm Differentiation

Since glucose and glutamine metabolism are essential for the maintenance of pluripotency in ICM and mouse and human PSCs, reduction of glucose and glutamine metabolism will affect the differentiation efficiency. Predictably, when mouse and human PSCs exit pluripotency, metabolism switches away from glycolysis ^(31), (35)^. In fact, during early differentiation from hPSCs, metabolic switching by MYC/MYCN is a critical step for germ layer specification. In hPSCs, stable expression of MYC/MYCN transcription is required for the maintenance of elevated glycolysis. In addition, each decline in MYC or MYCN expression is required for the transition of pluripotency to the mesoderm or definitive endoderm, resulting in metabolic switching from glycolysis to OXPHOS (Figure 2) ^(38)^. Furthermore, retinoic acid, an upstream negative regulator of MYC/MYCN, also induces differentiation from primed hPSCs, with decreased glucose and increased oxygen consumption ^(35)^. Mesodermal cells show a higher oxygen consumption rate (OCR) and lower extracellular acidification rate (ECAR) than hPSCs, ectoderm, and endoderm cells ^(39)^. Before differentiation, pyruvate supplementation, the main product of glycolysis upregulates the OCR/ECAR ratio and expression of mesodermal markers, and it induces mesodermal cell via activation of AMPK pathway ^(40)^. Researchers reported that insulin blocked mesendodermal differentiation and cardiogenesis from hPSCs by inhibiting the expression of Brachyury (T), Foxa2, Gata4, and Nkx2-5 ^(41), (42)^. Moreover, insulin is known to be associated with glycolysis. When insulin binds to its receptor in the cell membrane, it promotes recruitment of glucose transporters from cytoplasm to cell membrane, leading to increase in glucose uptake. This suggests that insulin depletion forces the metabolic changes from glycolysis to OXPHOS during cardiac differentiation. This metabolic change may be associated with the early mesodermal differentiation in mouse and human PSCs. In mesodermal induction, it is well known that the activation of canonical Wnt/β-catenin signaling is indispensable ^(3)^. Glycogen synthase kinase 3β (GSK3β) is involved in the phosphorylation of β-catenin, leading to degradation by proteasome. Therefore, GSK3β inhibitors (e.g. CHIR99021) activate Wnt/β-catenin signaling. Besides, GSK3 is originally known to affect glucose metabolism through insulin signaling ^(43)^. In short, GSK3 inhibits glycogen synthesis via phosphorylation of glycogen synthase (GS), which is a key enzyme to convert glucose to glycogen, inducing activated glycolysis.

The Hippo signaling pathway regulates the cell proliferation and organ size. Yes-associated protein (Yap) is one of the critical downstream effectors of the Hippo pathway, and it interacts with SMAD and Oct4. Yap-SMAD-Oct4 binds with NuRD repressor complex to maintain pluripotency and inhibit mesodermal differentiation of hPSCs via a decrease in Wnt3 expression ^(44), (45)^. In zebrafish liver and human liver cancer cells, Yap directly enhances glutamine synthetase expression and activates glutamine metabolism for nucleic acid synthesis ^(46)^. Furthermore, the regulation of glucose transporter 1 (Glut1) expression and glucose uptake by Yap is highly conserved in mammals ^(47)^. Therefore, reduction of Yap expression is essential for metabolic transition from glycolysis/glutamine metabolism to OXPHOS and important for mesodermal differentiation. Especially, most cancer cells mainly rely on the activated glycolysis via GSK3-mediated inhibition of GS even under normoxia, which is called the “Warburg Effect.” Hence, inhibition of activated glycolysis by restricting GSK3 function is considered a target of cancer therapeutics. Researchers also reported that GSK3 increased glycolytic flux to proliferate B cells when the antigen is encountered, indicating that GSK3 acts as a metabolic sensor to regulate immune response by regulating the glycolytic pathway ^(48)^. In skeletal muscle, inactivation of GSK3β increases fatty acid oxidation (FAO) via activation of PPAR-γ-co-activator1, a master regulator of mitochondrial biosynthesis ^(49)^. Taken together, the assumption exists that GSK3 inhibition induces a metabolic shift from glycolysis to OXPHOS during mesodermal differentiation.

## Metabolism in Differentiated Immature Cardiomyocytes

Although a developing heart mainly depends on OXPHOS compared to ICM and PSCs, metabolic changes also occur during heart development. As the embryonic and fetal hearts exist in hypoxic environment, they depend on glycolysis rather than OXPHOS to generate ATP (Figure 2). Glucose dependence of embryonic and fetal heart may be due to hypoxia inducible factor 1α (HIF-1α), a transcriptional factor activated by low oxygen. HIF-1α, a key regulator for glycolysis, regulates glycolysis-related genes, such as *glut1* and hexokinase1 (*hk1*), by directly binding to hypoxia response elements located on the upstream of HIF-1α target genes ^(50), (51)^. During heart development, *Nkx2-5*-specific deletion of HIF-1α causes hypoplasia and arrested cardiac development in mice ^(52)^. Moreover, *α-myosin heavy chain (αMHC)^Cre/+^-VHL* mice, which maintain stable expression of HIF-1α in normoxic condition, show low mitochondrial function and ATP generation, suggesting that the decreased HIF-1α signaling in cardiomyocytes after birth controls metabolic changes from glycolysis to OXPHOS ^(53)^. In fact, embryonic and fetal cardiomyocytes do not depend much on FAO under 15% of total ATP production ^(51)^. Conversely, a loss of mitochondrial transcription factor A (known as *tfam*) causes severe mtDNA depletion with abolished OXPHOS and leads to heart malformation and embryonic lethal prior to E10.5 in mice, suggesting that mitochondria also exhibit a pivotal role in heart development, because dependency on OXPHOS in embryonic and fetal hearts is still higher than that in ICM and early differentiated cells ^(54)^. Mitochondrial morphological changes are also important for cardiac differentiation in developing hearts (Figure 2). The mitochondrial permeability transition pore (mPTP) is open in embryonic hearts, while it is closed during development. Closed mPTP drives mitochondrial elongation and higher mitochondrial membrane potential in mouse hearts ^(55)^.

During cardiac differentiation from mPSCs, the basal mitochondrial respiration and respiratory capacity is significantly increased, whereas glycolysis is decreased ^(56)^. The expression levels of the genes involved in mitochondrial fusion and cristae maturation in differentiated cardiomyocytes are higher than those in mPSCs ^(56)^. Consistent with this, mouse and human PSC-derived cardiomyocytes show higher mitochondrial OXPHOS (Figure 2) ^(56)^. Inhibition of OXPHOS during cardiac differentiation causes reduction and abnormal translocation of mitochondria, leading to poor sarcomeric organization and a lower beating frequency ^(56)^. Moreover, as with a developing heart, mPTP inhibition by cyclosporine A also induces mitochondrial maturation and cardiac differentiation in mouse and human PSCs ^(57)^. These findings suggest that mitochondrial maturation and metabolic shift are necessary for cardiac differentiation from PSCs.

## Metabolism in Differentiated Mature Cardiomyocytes

Even immediately after birth, cardiomyocytes still rely on glycolysis and lactate oxidation (LO) as a source of ATP production. On day 7 after birth, glycolysis-dependency decreases to less than 10 % of total ATP production accompanied with increased dependence on LO and FAO in rabbit neonatal hearts (Figure 2) ^(50)^. Researchers reported that this metabolic change and cell cycle arrest through DNA damage response were affected by oxygen-rich environment ^(58)^. The dependence on LO dramatically decreases to less than 1% and that on FAO increases up to 80% of total ATP production, due to increased uptake of fatty acids for FAO in 21 days old rabbit neonatal hearts (Figure 2) ^(50)^.

It has been known that hPSC-derived cardiomyocytes are much less organized and smaller than the adult cardiomyocytes and resemble fetal cardiomyocytes, as described previously ^(59), (60)^. The condition of cardiac maturation is evaluated by structure, gene expression, energy, force, conduct, ion channel density, and Ca^2+^ kinetics ^(59)^. Understanding of heart developmental metabolism enables us to develop a method for obtaining matured and pure cardiomyocytes from hPSCs. Although hPSCs and hPSC-derived non-cardiac proliferating cells mainly depend on glucose and glutamine metabolism, differentiated immature cardiomyocytes rely on LO for ATP production (Figure 2) ^(12)^. By utilizing these metabolic differences, we successfully selected only cardiomyocytes under glucose- and glutamine-depleted but lactate containing conditions. Remarkably, this method forces a metabolic shift from glycolysis to LO and makes sarcomeric organization and mitochondria developed ^(14)^. Furthermore, metabolically selected PSC-derived cardiomyocytes enhance the ratio of OCR/ECAR by 1.8-fold compared to non-purified cardiomyocytes ^(61)^, suggesting that this method forces the metabolic maturation from embryonic to neonatal cardiomyocytes. These metabolic shifts of cardiomyocytes result into more mature action potentials and calcium handling with increased expression of cardiomyocyte-related ion channels and cardiac contraction genes ^(62)^. Researchers reported that activated glycolysis inhibited cardiac maturation through pentose phosphate pathway, which supplies NADPH, nucleotides, and lipids for proliferation. In contrast, glucose-depleted conditions promoted cardiac maturation with the increased expression of cardiac contract genes, functional beating, and morphological maturity *in vitro* ^(63)^. In addition, the efficiency of cardiac differentiation from mESCs is better in low glucose medium than in high glucose medium as high glucose medium suppresses the expression of mesoderm and cardiac transcription genes, resulting in the reduction of contractile cardiomyocytes ^(64)^. As one of the positive factors for cardiac maturation, researchers reported that palmitate administration accelerates cardiac maturation with highly organized sarcomeric structure, mechanical force, and gene expression profiles in cardiac organoids derived from hPSCs ^(65)^. Similar to postnatal hearts, the metabolic switching from glycolysis to FAO induces cardiac maturation through DNA damage response and cell cycle arrest (Figure 2). Furthermore, PGC1α, a major regulator of mitochondria, also plays a key physiological role in cardiac maturation by controlling ROS ^(66)^. According to the assessment of oxidative metabolism in hPSC-derived cardiomyocytes cultured in glucose containing media, glucose and fatty acid containing media, and fatty acid containing media, the fatty acid containing media enhanced oxidative metabolism and cardiac maturation, structurally and functionally ^(67)^. Genome-wide effects of fatty acid treatment showed that the genes involved in fatty acid transportation were upregulated, whereas the genes involved in glucose metabolism were downregulated ^(68)^. Moreover, extracellular matrix, providing essential scaffold for intercellular contact and signal molecules, is also a key regulator for cardiac maturation. mESC-derived cardiomyocytes, cultured on laminin-511/521, showed structural maturation and increased mitochondrial respiration ^(69)^. These findings indicate that both the intracellular metabolic profiles and mitochondrial function are critical for cardiac maturation as well as cardiac differentiation ^(58)^.

## Conclusion

Since the beginning of the learning of how to make a heart, researchers revealed that in addition to the network of transcriptional factors and epigenetic modifiers, metabolism is also dramatically changed in a spatiotemporal manner. The mechanisms of cardiac differentiation *in vitro *are highly correlated with the heart development *in vivo*; therefore, the methods of cardiac differentiation from PSCs are continuously improving in terms of differentiation efficiency and maturity. However, many issues such as variation among batches and lots are yet to be explored for an effective cardiac differentiation method. In addition, the same quality of differentiated cardiomyocytes should be obtained, physiologically and functionally. Furthermore, it is known that PSC-derived cardiomyocytes are more immature than adult cardiomyocytes *in vivo*, and a detailed understanding of the mechanisms for maturation of cardiomyocytes are also needed. In this review, we summarized the metabolic shift that characterized gene expression, structure, and function during cardiac differentiation. Comprehension among transcriptional network, signal pathway, and metabolism will give us breakthrough achievement. In the future, it may be realized that this knowledge on the metabolism in each cell type will give us better and correct understandings of the network of metabolic plasticity, gene regulation, and epigenetic modification. Furthermore, the metabolic feature may become an important criterion of differentiation, maturation, and disease state. If so, the metabolic information of the cells may be the factor to further ensure the safety of cell transplantation.

## Article Information

### 

This article is based on the study, which received the Medical Research Encouragement Prize of The Japan Medical Association in 2019.

### Conflicts of Interest

S. T. owns equity in Heartseed. Inc.

### Sources of Funding

This work was supported by the Medical Research Encouragement Prize of The Japan Medical Association (S. T.) and Projects for Technological Development, Research Center Network for Realization of Regenerative Medicine by Japan, the Japan Agency for Medical Research and Development grant (20bm0404023h0003 to S. T.), and partly supported by JSPS KAKENHI grant (19K22626 to S. T, 20J01097 to Y. M.). 

### Author Contributions

Y. M. and S. T. made substantial contributions to the conception and design of the work.

### Approval by Institutional Review Board (IRB)

This manuscript is a review and therefore did not need approval by an IRB.
